# The Significant Surface-Water Connectivity of “Geographically Isolated Wetlands”

**DOI:** 10.1007/s13157-017-0887-3

**Published:** 2017

**Authors:** Aram J. K. Calhoun, David M. Mushet, Laurie C. Alexander, Edward S. DeKeyser, Laurie Fowler, Charles R. Lane, Megan W. Lang, Mark C. Rains, Stephen C. Richter, Susan C. Walls

**Affiliations:** 1 Department of Wildlife, Fisheries, and Conservation Biology, University of Maine, Orono, ME 04469, USA; 2 Northern Prairie Wildlife Research Center, US Geological Survey, 8711 37th Street SE, Jamestown, ND 58401, USA; 3 Office of Research and Development, US Environmental Protection Agency, 1200 Pennsylvania Ave NW (8623-P), Washington, DC 20460, USA; 4 School of Natural Resource Sciences, North Dakota State University, Fargo, ND 58108, USA; 5 School of Law, University of Georgia, Athens, GA 30602, USA; 6 Office of Research and Development, US Environmental Protection Agency, Cincinnati, OH 45268, USA; 7 National Wetland Inventory, US Fish and Wildlife Service, Falls Church, VA 22041, USA; 8 School of Geosciences, University of South Florida, Tampa, FL 33620, USA; 9 Department of Biological Sciences and Division of Natural Areas, Eastern Kentucky University, Richmond, KY 40475, USA; 10 Wetland and Aquatic Research Center, US Geological Survey, 7920 NW 71st Street, Gainesville, FL 32653, USA

**Keywords:** Clean Water Act, Connectivity, Geographic isolation, Hydrology, Streams, Upland embedded wetlands, Waters of the U.S.

## Abstract

We evaluated the current literature, coupled with our collective research expertise, on surface-water connectivity of wetlands considered to be “geographically isolated” (*sensu* Tiner Wetlands 23:494–516, 2003a) to critically assess the scientific foundation of grouping wetlands based on the singular condition of being surrounded by uplands. The most recent research on wetlands considered to be “geographically isolated” shows the difficulties in grouping an ecological resource that does not reliably indicate lack of surface water connectivity in order to meet legal, regulatory, or scientific needs. Additionally, the practice of identifying “geographically isolated wetlands” based on distance from a stream can result in gross overestimates of the number of wetlands lacking ecologically important surface-water connections. Our findings do not support use of the overly simplistic label of “geographically isolated wetlands”. Wetlands surrounded by uplands vary in function and surface-water connections based on wetland landscape setting, context, climate, and geographic region and should be evaluated as such. We found that the “geographically isolated” grouping does not reflect our understanding of the hydrologic variability of these wetlands and hence does not benefit conservation of the Nation’s diverse wetland resources. Therefore, we strongly discourage use of categorizations that provide overly simplistic views of surface-water connectivity of wetlands fully embedded in upland landscapes.

## Introduction

Throughout the world, small wetlands with seasonal hydrology are at great risk of loss or degradation and effective approaches to conserving their functions lag behind the increase in threats (Calhoun et al. 2016). For this reason, researchers and managers need to improve the understanding of vulnerable wetland functions and this includes both continuing research and clarifying regulations that do exist while considering alternative approaches. In this paper, we give one example of addressing this issue that has relevance to wetland managers globally. In a recent issue of *WETLANDS*, we published an essay entitled “Geographically Isolated Wetlands: Rethinking a Misnomer” (Mushet et al. 2015). In our paper, we described the declining relevance and confusing nature of the “geographically isolated wetlands” (GIWs) categorization as currently used in wetland science and policy in the United States. Leibowitz (2015) published a thoughtful response to our review in which he defended the use of the categorization and argued that there are important scientific, legal, and regulatory needs for identifying wetlands that are completely surrounded by uplands (i.e., GIWs, sensu Tiner 2003a). We have found that scientific responses to the legacies of the last decade’s court actions and policy needs for wetland regulation under the Clean Water Act (CWA; 33 U.S.C. Chapter 26) have improved our understanding of the complexity of wetland hydrology, functions, and nexuses that transcend simple assessments of degree of upland embeddedness. Grouping wetlands by whether or not they are surrounded by uplands does not indicate a lack of a “significant nexus,” and therefore does not provide a useful separation for meeting legal and regulatory information needs (Cohen et al. 2016).

We provide a brief review of key scientific findings to instantiate our thesis that having a static category based on upland embeddedness is no longer beneficial and, in fact, may be detrimental to conservation of these wetland resources and their influence on downgradient systems. The GIW term, or any term that implies that wetlands surrounded by uplands are in fact functionally isolated, is difficult to justify scientifically, difficult to apply pragmatically, subject to misuse and misinterpretation, and maps poorly onto the regulatory landscape. In this paper, we use the term upland-embedded wetland to describe a geospatial setting with no assumptions about connectivity or lack thereof and with no intent to replace the GIW term with “upland-embedded wetland”. We focus on surface-water connections, as the GIW categorization has not been promoted as providing meaningful insights into other forms of connectivity (e.g., groundwater, biogeochemical, biotic) that clearly transcend degrees of upland embeddedness. We define surface water connectivity as flow of surface water (episodic, seasonal, or semi-permanent) between two unique landscape elements that may or may not be linked by an aquatic feature with a bed and bank (i.e., channel or other indicators of flow permanence).

## Dynamic Surface-Water Connections

Upland-embedded wetlands occur along continuous spatial and temporal gradients, from highly connected to highly disconnected (Cohen et al. 2016). Research on upland-embedded wetlands demonstrates that many have surface-water connections to other aquatic landscape components (e.g., rivers, streams, lakes, other wetlands; Vanderhoof et al. 2016). A brief synthesis of key findings in the literature follows.

A conceptual model for thinking about how upland-embedded wetlands function at broader ecosystem scales is provided by Rains et al. (2016). They describe upland embedded wetlands as nodes in hydrological networks and state that these wetlands are “…integrally connected to uplands, other wetlands, and downgradient waters.” The authors further describe complex lag, sink, and source functions of these wetlands and their resultant influences on surface-water and shallow-groundwater flows to downgradient waters (also see Golden et al. 2016). Rains et al. (2016) describe a wide range of surface-water connectivity displayed by wetlands, with wetlands identified as GIWs ranging from “infrequent/absent surface connectivity” (i.e., isolated) to “intermittent surface connectivity” (i.e., clearly not isolated). Likewise, Leibowitz (2015) describes GIWs that range from a wetland connected to a river by surface flow through a non-channelized swale to a geographically isolated wetland that is hydrologically isolated from a river. The key feature of the continuous range of surface-water connectivity described by both Leibowitz (2015) and Rains et al. (2016) is magnitude and timing, not the degree to which a wetland is surrounded by upland. While the “isolated” term has been used to describe the surface connections of all upland-embedded wetlands, the term describes only a subset of GIWs.

Consider work in the Prairie Pothole Region (PPR) of the Midwestern USA. Wetlands in this region have long been iconic examples of “geographically isolated” wetlands (Tiner 2003a) yet current research has documented high levels of hydrologic, biologic, and biogeochemical connectivity (Marton et al. 2015; Mushet et al. 2015; Cohen et al. 2016; Leibowitz et al. 2016; McLean et al. 2016). For example, Leibowitz et al. (2016) described the complex spill-and-fill and spill-and-merge surface-water connectivity of eight prairie pothole wetlands over a 26-year period (1979–2015). Their findings suggest that research exploring the effects of surface-water connections needs to address the specific types of connections and not broader categories. Further, in a detailed analysis of wetland hydrology, Hayashi et al. (2016) demonstrated how Midwestern USA prairie pothole wetlands and their upland catchments function as integrated units whose existence depends on the lateral movement of both subsurface and surface runoff water. Furthermore, they found that differences in surface-water connectivity among individual wetlands controlled ponded-water permanence, leading to a diversity of wetland functional types within wetland complexes.

The importance of surface-water connections to many wetlands considered to be “geographically isolated” is also supported by research that has documented high levels of hydrologic, biologic, and biogeochemical connectivity among vernal pools in California, USA (Golden et al. 2016; Rains et al. 2016). Western vernal pools are small depressional wetlands commonly connected by swales to one another and downgradient waters. The climate of this landscape is Mediterranean with pronounced wet and dry seasons. In the dry season, the variable source area from which streamflow is derived contracts and vernal pools may present as upland or upland-embedded wetlands. However, in the wet season, these vernal pools and swales become part of the river network system. These surface-water connections are not speculative or insubstantial, with measured surface-water connections for as many as 150–200 days being reported (Rains et al. 2006; Rains et al. 2008).

**Fig. 1:**
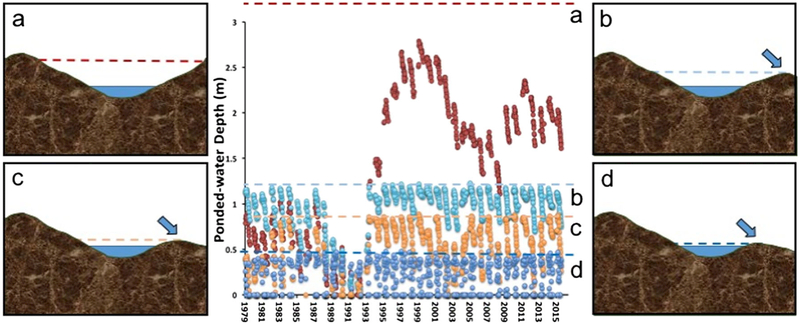
Little knowledge about magnitude and timing of surface-water connectivity is gained by knowing that a wetland is surrounded by upland, i.e., is “geographically isolated.” The above hydrograph displays water levels of four “geographically isolated” prairie-pothole wetlands (labeled a–d) at the Cottonwood Lake Study Area in Stutsman County, North Dakota, over a 36-year period (1979–2015). The drawings on the left and right of the hydrograph characterize the upland-embedded basins of the wetlands. External spill points (arrows), as defined by Leibowitz et al. (2016), set limits (color-coded dashed lines) to water storage and thus the magnitude of water losses from these wetland basins. Wetland P1 (**a**) is situated within a deep basin that does not have a realized external spill-point and thus does not contribute (i.e., spill) to down-gradient surface-water flows. By contrast, wetlands P8 (**b**), P3 (**c**), and T6 (**d**) each, to varying degrees, contribute to down-gradient flows when water levels reach an external spill point. The magnitude and timing of these surface-water flows vary greatly with similarly variable hydrological, geochemical and ecological effects

The condition of being wholly embedded within an upland matrix does not reliably indicate lack of surface water connectivity to other aquatic ecosystems. In short, there is a continuum of connectivity that applies to an individual wetland, complexes of wetlands, and wetlands within an ecoregion. Furthermore, abiotic factors including soil type, precipitation patterns and geomorphology are often significant factors influencing degrees and nature of surface water connections, but these factors are not accounted for by the label “geographically isolated” (Fig. 1).

## Distance as a Surrogate for Isolation

A reexamination of the commonly used practice of identifying upland-embedded wetlands based on distance from a stream or large water body reveals that this methodology may result in a gross overestimate of the number of wetlands lacking significant surface flows to downstream waters (i.e., the condition that the GIW designation is assumed to identify). Vanderhoof et al. (2016) found notable variation among ecoregions in empirically measured distances at which wetlands connected via surface water to mapped streams, making it problematic to identify surface-water connected wetlands based on distance alone. For example, in the Des Moines Lobe ecoregion of the PPR, the authors found that 78% of surface-water connected wetlands were located within 400 m of a mapped stream. However, in the Drift Plains ecoregion of the PPR, only 52% of the connected wetlands were located within that same stream-buffer distance. Relative to these findings, most buffer distances previously used to identify upland-embedded wetlands (e.g., 76 m, Levin et al. 2002; 20–40 m, Tiner et al. 2002 and Tiner 2003b; 10 m, Frohn et al. 2009; 10 m, Reif et al. 2009; 20 or 40 m buffer for small streams and 300 m for large streams, Vance 2009; 10 m, Lane et al. 2012; 10 m, Lane and D’Amico 2016) are likely insufficient to judge surface water connectivity within some landscapes. As a result, numerous surface-water connected wetlands located beyond the threshold buffer distance are being grouped with wetlands lacking such connections. Not surprisingly, Lane and D’Amico (2016) found that increasing their 10-m buffer distance to 300 m resulted in a significant decrease in the number of putative GIWs in multiple ecoregions across the US. Further, Golden et al. (2016) found in their modeling assessment of the influence of GIWs on downgradient streamflow in the lower Neuse River Basin, North Carolina, USA, that the farther upland-embedded wetlands were located from downgradient streams, the greater their potential contributions to streamflow across long time scales (i.e., seasonally and annually). With the inclusion of all wetlands in the analyses, this effect disappeared. Therefore, many quantifications of upland-embedded wetlands likely have overestimated occurrence of non-connected wetlands since the multitude of connected wetlands outside the buffer distance are identified as isolated. Furthermore, Lang et al. (2012) found that commonly available stream vector datasets (e.g., the US Geological Survey National Hydrography Dataset [NHD]) used to quantify wetland–stream connections underestimate stream length, at least in relatively wet regions like the eastern US. This is partially explained by the fact that the NHD dataset was not designed to map ephemeral streams or streams < 1.6 km in length. Lang et al. (2012) concluded that these factors would lead many wetlands to be incorrectly considered to be disconnected from the stream network. This is counter to arguments that quantifications derived using buffers are conservative estimates of the numbers of upland-embedded wetlands (Leibowitz 2015).

Direct pre-identification of upland-embedded wetlands will continue to lessen the guesswork currently employed in establishing regulatory connectivity. For example, Wu and Lane (2016) developed a new approach to identifying wetland depressions in the PPR that accounts for dynamic filling, spilling and merging hydrological processes not considered in previous algorithms designed to identify such depressions (Leibowitz et al. 2016). Even low-tech methods involving using local knowledge and ground-truthing involving citizen-scientists can produce important information on current pools and past occurrences of connectivity. Levesque et al. (2016) describe a vernal pool conservation initiative in New England, USA, that recognizes the landscape-scale functions of vernal pools and encourages conservation of “poolscapes” in partnership with land trusts and other conservation groups who recognize the value of conserving ecosystem connections—work all driven by community based collaboration.

Proximity to mapped streams and other drainage features have been used as proxies for surface water connectivity (see above) because of the difficulty inherent in quantifying surface-water connectivity (Lane and D’Amico 2016). More advanced technologies and approaches provide promising solutions to better characterize connectivity. For example, other methods that could be examined include direct monitoring of inundation patterns using lidar intensity, multispectral and synthetic aperture radar data, predicting flow based on slope derived from lidar-based digital elevation models, and using process-based hydrologic models parameterized using geospatial data. Methodologies that move away from a categorical definition of geographically isolated wetlands and more closely approximate the adjacent versus non-adjacent definition will be better aligned with current legal/regulatory needs.

## Legal/Regulatory Considerations

In the years immediately following the U.S. Supreme Court’s 2001 decision in *SWANCC* (*Solid Waste Agency of Northern Cook County* [*SWANCC*] vs. *US Army Corps of Engineers, 531 US* 159), there was a great deal of confusion regarding the concept of an “isolated” wetland. In scientific literature, this term was commonly used to describe various types of depressional wetlands (e.g., Damman and French 1987; Semlitsch and Bodie 1998; Bailey 1999). Following scientific usage, the Corps of Engineers promulgated a regulatory definition of “isolated wetland” for administration of their Nationwide Permit Program (NWP) 26 (33 CFR 330.2(e)). Prior to *SWANCC*, neither usage was relevant to Clean Water Act (CWA) jurisdiction (Downing et al. 2003). Following the *SWANCC* and later *Rapanos* (*Rapanos* vs. *United States*, *547 U.S. 715,2006* decisions), existing definitions were muddied by case law that misinterpreted scientific and regulatory concepts of “isolation” and “adjacency” as end-members of waterbody functional connectivity. At its inception, the term “geographically isolated wetland” was meant to correct this misinterpretation and avoid further error (e.g., Tiner et al. 2002; Leibowitz 2003; Tiner 2003a). Unfortunately, the clarification presented in those seminal publications warning that geographic isolation should not be used to infer functional isolation did not communicate well to other communities of practice (e.g., evolution, ecology, genetics) in which “geographic isolation” has a very specific functional definition (Mushet et al. 2015). The science now shows that the degree of wetland surface-water connectivity cannot be assessed in a meaningful way by a simple determination of upland embeddedness (USEPA 2015; Rains et al. 2016; Cohen et al. 2016).

The recent Clean Water Rule (CWR, 80 FR 37054), which is currently stayed, does not use the GIW term, suggesting that federal agencies have moved beyond consideration of “geo-graphic isolation” as a factor for determining CWA jurisdiction. Instead, the rule recognizes the best-available science by establishing five subcategories of wetlands (prairie potholes, Carolina and Delmarva bays, pocosins, western vernal pools in California, and Texas coastal prairie wetlands) that must be considered as “similarly situated” (that is, functioning as systems at the water-shed scale) rather than as individual wetland basins, when determining their influence on navigable waters (CWR, 80 FR 37054). This consideration of the watershed-scale cumulative effects of wetlands and wetland complexes rather than individual basins is a large step forward from the localized, basin-scale assessments inherent in GIW categorization (Tiner 2003a; and Leibowitz 2015).

## Conclusions

Recent research findings show that wetlands surrounded by uplands vary greatly in occurrence, type, as well as frequency, timing, and importance of surface-water connections to other aquatic systems (Rains et al. 2016; Cohen et al. 2016). Ambiguous generalizations about degrees of connectivity and isolation between upland-embedded wetlands and other wetlands and downstream waters are illogical (Mushet et al. 2015). The single condition of being surrounded by uplands currently used by wetland scientists to define “geographic isolation” does not provide a useful separation between wetlands that have a significant surface-water connection and those that do not. Upland embeddedness does not necessarily provide any indication that these wetlands are functionally “isolated”.

Current research on connectivity of wetlands to downstream waters clearly shows that scientific needs are best met when gradients of surface-water connectivity are considered rather than through the use of a grouping defined by a threshold that does not reliably separate surface water connected/isolated wetlands, yet alone functionally connected/isolated wetlands. Embracing this knowledge requires a rethinking of our use of the “geographically isolated wetlands” misnomer and opens up advanced avenues to conserving wetland landscapes. Fully embracing the scientific knowledge gained since inception of the GIW grouping, knowledge that has identified the inherent connectedness of these “isolated” wetlands individually and as complexes, is needed to facilitate the long-term conservation of these important, and increasingly threatened, wetland resources. Conservation decisions cannot be made based on a broad category that, while created to help alleviate confusion over the term “isolated,” has instead further muddied the waters. Recognizing the diverse functions supported by gradients of wetland connectivity will lead to better conservation of all wetland resources.
